# Association of Peripheral Vestibular Disorder with Diabetes: A Population-Based Study

**DOI:** 10.3390/jpm14070768

**Published:** 2024-07-19

**Authors:** Tzong-Hann Yang, Chao-Hung Chen, Yen-Fu Cheng, Herng-Ching Lin, Chin-Shyan Chen

**Affiliations:** 1 Department of Otorhinolaryngology, Taipei City Hospital, Taipei 100, Taiwan; dad64@tpech.gov.tw; 2 Department of Speech, Language and Audiology, National Taipei University of Nursing and Health, Taipei 112, Taiwan; 3 Department of Otolaryngology-Head and Neck Surgery, School of Medicine, National Yang Ming Chiao Tung University, Taipei 112, Taiwan; fcheng2@vghtpe.gov.tw; 4 Center of General Education, University of Taipei, Taipei 111, Taiwan; 5 Research Center of Data Science on Healthcare Industry, College of Management, Taipei Medical University, Taipei 110, Taiwan; 6 Department of Thoracic Surgery, Mackay Memorial Hospital, Taipei 104, Taiwan; chchen@mmh.org.tw; 7 Department of Medicine, MacKay Medical College, New Taipei City 252, Taiwan; 8 Department of Medical Research, Taipei Veterans General Hospital, Taipei 112, Taiwan; 9 Department of Otolaryngology-Head and Neck Surgery, Taipei Veterans General Hospital, Taipei 112, Taiwan; 10 School of Health Care Administration, College of Management, Taipei Medical University, Taipei 110, Taiwan; 11 Research Center of Sleep Medicine, Taipei Medical University Hospital, Taipei 110, Taiwan; 12 Department of Economics, National Taipei University, New Taipei City 237, Taiwan

**Keywords:** vertigo, dizziness, diabetes, peripheral vestibular disorder, Ménière’s disease, benign paroxysmal positional vertigo

## Abstract

Background: This study aimed to investigate the association of peripheral vestibular disorders with type 1 and type 2 diabetes using a population-based dataset. Methods: The data for this study were obtained from Taiwan’s Longitudinal Health Insurance Database 2010. The sample consisted of 150,916 patients who were newly diagnosed with peripheral vestibular disorders as cases and 452,748 propensity-score-matching controls without peripheral vestibular disorders. We utilized multivariate logistic regression models to quantitatively evaluate the association between peripheral vestibular disorders and diabetes while considering factors such as sex, age, geographic location, monthly income, urbanization level of the patient’s residence, coronary heart disease, hypertension, and hyperlipidemia. Results: The chi-squared test indicates that diabetes was more common in the peripheral vestibular disorder group compared to controls (20.6% vs. 15.1%, *p* < 0.001). Of all sampled patients, the adjusted odds ratio for diabetes was 1.597 (95% CI = 1.570~1.623) for those with peripheral vestibular disorders when compared to controls, while patients with Ménière’s disease, benign paroxysmal positional vertigo, unilateral vestibulopathy, and other peripheral vestibular disorders had respective adjusted odds ratios of diabetes at 1.566 (95% CI = 1.498~1.638), 1.677 (95% CI = 1.603~1.755), 1.592 (95% CI = 1.504~1.685), and 1.588 (95% CI = l.555~1.621) in comparison to controls. Conclusions: Our research has revealed an association between diabetes and an increased susceptibility to peripheral vestibular disorders.

## 1. Introduction

Diabetes mellitus encompasses a spectrum of metabolic disorders marked by high blood sugar levels, stemming from issues in insulin secretion or action. Classified into four types, Type 1 Diabetes Mellitus (T1DM) involves autoimmune destruction of β cells and accounts for less than 10% of cases, while Type 2 Diabetes Mellitus (T2DM), comprising over 90% of cases, features insulin resistance and subsequent β cell failure. Other forms arise from genetic mutations, pancreatic anomalies, or endocrinopathies, and gestational diabetes develops during pregnancy. Presently, diabetes affects more than half a billion people globally, over 10.5% of the adult population, with projections indicating an increase to 12.2% by 2045 [1], mirroring the upward trend observed in Taiwan [2].

Persistent hyperglycemia significantly damages blood vessels, impairing endothelial function and leading to both macrovascular complications like atherosclerosis and microvascular issues such as retinopathy and peripheral neuropathy. This damage can further compromise the vestibular pathways, including central vestibular pathways due to vascular encephalopathy and peripheral vestibular pathways. These conditions are known to impair balance and increase the risk of falls [3]. While vascular encephalopathy is not directly linked to peripheral vestibular disorders, it is a significant risk factor for central vestibular disorders. This relationship is crucial because it affects peripheral vestibular disorders by disrupting central vestibular compensation. Falls, a severe consequence of impaired balance, become even more dangerous with vestibular dysfunction, a recognized risk factor for falls [4]. Despite managing peripheral neuropathy and retinopathy, vestibular dysfunction alone increases the likelihood of falling by over twice in diabetic patients. During the acute phase of peripheral vestibular disorders, vertigo is the primary symptom, but dizziness can also occur outside this phase. These findings underscore the importance of addressing vestibular dysfunction as part of comprehensive diabetes care to reduce fall risk and improve overall balance.

Peripheral vestibular disorders encompass a wide range of perceptions, such as illusions of self-motion, environmental motion, or a sense of swaying and tilting. The majority (80%) of vertigo cases arise from peripheral vestibular dysfunction, with benign paroxysmal positional vertigo, unilateral vestibulopathy (previously known as vestibular neuritis), and Ménière’s disease being the most prevalent [5]. For over a century, researchers have investigated the link between diabetes mellitus and the peripheral vestibular system. While previous animal studies explored vestibular deficits in diabetes, human temporal bone pathophysiology studies examined vestibular impairments in diabetic patient [6]. Research has also delved into various aspects of vestibular function using methods like vestibular evoked myogenic potentials, video head impulse tests, modified Romberg tests, and caloric tests [3,4]. Furthermore, investigations have explored the relationship between diabetes mellitus and specific peripheral vestibular disorders like benign paroxysmal positional vertigo, unilateral vestibulopathy, and Ménière’s disease [7,8,9,10], but these were limited to case series, hospital-based samples, or surveys. A dearth of large-scale population-based epidemiological studies examining the association between diabetes mellitus and peripheral vestibular disorders remains, including Ménière’s disease, unilateral vestibulopathy, benign paroxysmal positional vertigo, and other unspecified peripheral vestibular dizziness. Therefore, this study aims to bridge this knowledge gap by utilizing medical care claims data from Taiwan’s National Health Insurance (NHI) system to determine said relationship.

## 2. Materials and Methods

### 2.1. Database

The retrospective observational study relied on a sample extracted from Taiwan’s Longitudinal Health Insurance Database 2010 (LHID2010), which comprises the medical claims and registration files of two million beneficiaries enrolled in the NHI program, a universal coverage, single-payer system that has been available to all citizens since 1995. The LHID2010 was drawn randomly from the year 2010 Registry of NHI Beneficiaries, and, according to the National Health Research Institutes of Taiwan, it is a representative cross-section of the NHI beneficiary population regarding gender, age, and average payroll-related insurance deductions. The Registration files of the NHI dataset include registry for board-certified specialists, registry for medical personnel, registry for catastrophic illness patients, registry for contracted beds, registry for contracted specialty services, registry for contracted medical facilities, registry for medical services, registry for drug prescriptions, and registry for beneficiaries according to the NHI dataset introductions from the National Health Research Institutes of Taiwan. Furthermore, the original claim data of the NHI dataset consist of monthly claim summaries for ambulatory care claims, inpatient expenditures by admissions, details of inpatient orders, ambulatory care expenditures by visits, monthly claim summaries for inpatient claims, details of ambulatory care orders, expenditures for prescriptions dispensed at contracted pharmacies, and details of prescriptions dispensed at contracted pharmacies. Deidentified data from this database have been utilized by numerous Taiwanese researchers for epidemiological and clinical care research purposes. Researchers seeking access to the National Health Insurance (NHI) dataset in Taiwan are obligated to sign a formal agreement. This agreement ensures that researchers commit to respecting the confidentiality and privacy of patients and healthcare providers by not attempting to access personally identifiable information. This safeguard is crucial for maintaining trust in the healthcare system and adhering to ethical research practices.

This research was sanctioned by the institutional review board of Taipei Medical University (TMU-JIRB N202311011) and adheres to the Declaration of Helsinki. As we utilized deidentified administrative data, patient informed consent was waived by the institutional review board of Taipei Medical University.

### 2.2. Identification of Study Patients

For the purpose of case selection in this study, we identified a total of 150,916 individuals diagnosed with peripheral vestibular disorders using ICD codes. These diagnoses included Ménière’s disease (ICD-9-CM 386.0 or ICD-10-CM H81.0), benign paroxysmal positional vertigo (ICD-9 386.11 or ICD-10 code H81.10), unilateral vestibulopathy (ICD-9 386.12 or ICD-10 H81.2), and other/unspecified forms of peripheral vestibular disorders such as dizziness originating from the peripheral vestibular system, which were coded under ICD-9 386.10 (peripheral vertigo, unspecified), 386.19 (other aural vertigo or otogenic vertigo), and/or 386.9 (unspecified vertiginous syndromes and labyrinthine disorders) and under ICD-10 codes H81.31 (aural vertigo), H81.39 (other peripheral vertigo), H81.9 (unspecified disorder of vestibular function), and/or H83 (labyrinthitis). We limited our sample to those individuals who received their first-time diagnosis between 1 January 2012 and 31 December 2019—using the date they received their initial peripheral vestibular disorders diagnosis as our index date for further analysis purposes.

In order to assess the correlation between peripheral vestibular disorders and diabetes mellitus, we utilized a propensity-score-matching approach to select controls from the remaining beneficiaries aged 20 years and above in the registry of beneficiaries of LHID2010. Any enrollees with previous medical claims for peripheral vestibular disorders were excluded. Propensity scores were computed for all selected patients (150,916 with peripheral vestibular disorders and remaining beneficiaries) using logistic regression models that adjusted for age, sex, monthly income (NT)$0~15,840, NT$15,841~25,000, or ≥NT$25,001; US$1 ≈ NT$28 in 2021), geographic location (Northern, Central Southern, and Eastern regions), urbanization level of patient’s residence (5 levels where level 1 represents most urbanized while level 5 represents least urbanized), as well as coronary heart disease, hypertension, and hyperlipidemia. Subsequently, each sampled patient with peripheral vestibular disorders was matched with three controls without peripheral vestibular disorders through the nearest neighbor random matching algorithm along caliper adjustment using priori value +/−0.02. For cases, we assigned year of index date as year when first diagnosis of peripheral vestibular disorders was received, while, for controls, it was simply matched year during which they had an ambulatory care visit. The study sample thus comprised 150,916 patients who had been diagnosed with peripheral vestibular disorders alongside 452,748 control subjects who did not have this condition.

### 2.3. Measures of Outcomes

DM cases were discerned by means of ICD-9-CM code 250 or ICD-10-CM codes E10–E14. This inquiry solely incorporated DM cases that had been diagnosed with the ailment prior to the index date at least once.

### 2.4. Statistical Analysis

All statistical analyses were conducted using the SAS system (SAS System for Windows, vers. 9.4, SAS Institute, Cary, NC, USA). We utilized chi-square tests and *t*-tests to examine variations in baseline characteristics between patients with peripheral vestibular disorders and controls. Additionally, we employed multivariate logistic regression models to quantitatively assess the association of peripheral vestibular disorders with diabetes mellitus while considering factors such as sex, age, geographic location, monthly income, urbanization level of the patient’s residence, coronary heart disease, hypertension, and hyperlipidemia. Estimated odds ratios (ORs) alongside their corresponding 95% confidence intervals (CIs) were used to measure differences in odds of diabetes mellitus between patients with peripheral vestibular disorders and controls. All *p*-values were two-sided; a *p*-value < 0.05 was deemed statistically significant.

## 3. Results

The study sample consisted of 603,664 patients with a mean age of 56.4 ± 16.9 years (±standard deviation). After using the propensity-scored method to match patients with peripheral vestibular disorders and controls, Table 1 shows that there were no significant differences in terms of age (*p* = 0.653) and sex (*p* > 0.999). Of the sampled patients, only 38.6% were male. However, there were statistically significant differences in urbanization level (*p* = 0.003), monthly income (*p* = 0.002), and geographic region (*p* < 0.001) distribution between patients with peripheral vestibular disorders and controls. Patients with peripheral vestibular disorders were more likely to reside in the less urbanized communities (urbanizations levels 4 and 5) and communities located in the Eastern part of Taiwan relative to controls. In addition, patients with peripheral vestibular disorders had a greater tendency to have monthly income in the category of NT$15,841~25,000 compared to controls. However, it is important to note that large sample sizes may produce significant *p*-values even when differences are negligible in magnitude or composition; therefore, we further measured effect sizes by calculating Cohen’s h and found that the value of Cohen’s φ were all less than 0.01, indicating negligible significance between groups regarding the distributions of urbanization level, monthly income, and geographic region.

Furthermore, our findings revealed no statistically significant differences in prevalence rates for coronary heart disease (*p* > 0.999), hypertension (*p* > 0.999), and hyperlipidemia (*p* > 0.999) among patients with peripheral vestibular disorders compared to controls. Of the 603,664 sampled patients, 19.5%, 44.2%, and 42.5% had received the diagnoses of coronary heart disease, hypertension, and hyperlipidemia, respectively.

Table 2 depicts the prevalence of diabetes mellitus among the examined patients. The chi-squared test indicates a notable contrast in diabetic occurrence between individuals with peripheral vestibular disorders and those in the control group (20.6% vs. 15.1%, *p* < 0.001). Diabetes was more common in the peripheral vestibular disorder group compared to the control group. Furthermore, our findings reveal significant statistical differences in diabetes mellitus incidence among patients with Ménière’s disease (22.5% vs. 15.1%, *p* < 0.001), benign paroxysmal positional vertigo (20.7% vs. 15.1%, *p* < 0.001), unilateral vestibulopathy (20.6% vs. 15.1, *p* < 0.001), as well as other peripheral vestibular disorders (20.2% vs. 15.1, *p* < 0.001) relative to controls.

Table 3 displays the ORs of diabetes mellitus among patients with peripheral vestibular disorders in comparison to controls. The ORs for diabetes mellitus amongst sampled patients with peripheral vestibular disorders, Ménière’s disease, benign paroxysmal positional vertigo, unilateral vestibulopathy, and other peripheral vestibular disorders were 1.463 (95% CI = 1.441~1.485), 1.443 (95% CI = 1.387~1.502), 1.522 (95% CI = 1.462~1.585), 1.466 (95% CI = 1.393~1.543), and 1.454 (95% CI = 1.428~1.482), respectively, relative to the control group. In addition, after adjusting for age, sex, monthly income, geographic location, urbanization level of the patient’s residence, coronary heart disease, hypertension, and hyperlipidemia, the OR for diabetes mellitus amongst sampled patients with peripheral vestibular disorders was found to be 1.597 (95% CI = 1.570~1.623). Furthermore, after making similar adjustments for Ménière’s disease, benign paroxysmal positional vertigo, unilateral vestibulopathy, and other peripheral vestibular disorders cases, we discovered that adjusted ORs of diabetes mellitus were, respectively, recorded as 1.566 (95% CI = 1.498~1.638), 1.677 (95% CI = 1.603~1.755), 1.592 (95% CI = 1.504~1.685), and finally as 1.588 (95% CI =1.555 ~1.621).

## 4. Discussion

This study presents the initial widespread population-based inquiry into the interrelation between peripheral vestibular disorders and diabetes mellitus. Our discoveries unambiguously exhibit a noteworthy correlation between diabetes mellitus and peripheral vestibular disorders, encompassing Meniere’s disease, benign paroxysmal positional vertigo, unilateral vestibulopathy, and other peripheral vestibular disorders. By employing an exquisitely crafted propensity-score-matched case-control study, we have divulged compelling outcomes. Persons with diabetes display a 1.597-fold increased probability of developing peripheral vestibular disorders. Correspondingly, there is a notable increase in the probability of encountering benign paroxysmal positional vertigo, unilateral vestibulopathy, Meniere’s disease, and other peripheral vestibular disorders by 67.7%, 59.2%, 56.6% and 58.8%, respectively, for individuals with diabetes compared to their counterparts who do not have this condition. These significant findings provide valuable insights into the complex interplay between diabetes mellitus and peripheral vestibular disorders, thereby opening avenues for future targeted interventions aimed at gaining a deeper understanding of this association.

### 4.1. Diabetes Mellitus Is Associated with Ménière’s Disease

Our research has yielded noteworthy discoveries regarding the correlation between diabetes mellitus and Ménière’s disease. Despite a dearth of literature on this matter, with most studies being published 15 years ago, our study provides valuable elucidation to the field. A multitude of authors have documented anomalous carbohydrate metabolism and hyperinsulinemia in patients afflicted with Ménière’s disease, which is congruent with our own findings [11,12]. In a case series involving 574 patients with Ménière’s disease, 26% of them also suffered from diabetes mellitus [13]. Moreover, diabetes may influence the progression of Ménière’s disease, leading to more severe hearing impairment and more frequent vertigo [14,15] Additionally, when treating hearing loss, it was observed that patients without diabetes responded more successfully to treatment, while those with diabetes experienced more frequent hearing impairment. Furthermore, the analysis revealed that diabetes had a significant impact on the required dosage of the conservative therapy, betahistine-dihydrochloride, with patients with diabetes requiring higher daily doses [15]. However, it is worth noting that some studies have found no association between Meniere’s disease and diabetes mellitus [16,17]. Numerous studies have demonstrated that both type 1 and type 2 diabetes exert detrimental effects on the function of the vestibular and auditory systems within the inner ear, as observed in both human subjects and animal models [6,18]. Although the underlying mechanisms remain unidentified, studies suggest that the inner ear is a direct target for insulin action and insulin resistance [10,19]. Magnetic resonance imaging (MRI) studies have revealed that in mice fed a high-fat diet, a model for insulin resistance and diabetes [20], develop endolymphatic hydrops [10], supporting this theory. Additionally, the diagnosis of Ménière’s disease is mainly determined by identifying particular symptoms, which include two or more episodes of vertigo lasting between 20 min and 12 h, sensorineural hearing loss in the low to medium-frequency range, and fluctuating aural symptoms such as hearing alterations, tinnitus, and a feeling of fullness in the affected ear [21]. Interestingly, diabetes mellitus can also present with fluctuating hearing loss [11], tinnitus [7], and vertigo [3], making it possible for patients with diabetes to exhibit Ménière’s syndrome-like symptoms in our study. These findings highlight the intricate relationship between diabetes mellitus and Ménière’s disease, emphasizing the need for further investigation in this area.

### 4.2. Diabetes Mellitus Is Associated with Benign Paroxysmal Positional Vertigo

Our research provides evidence of a correlation between diabetes mellitus and benign paroxysmal positional vertigo. This discovery is consistent with previous studies that have also demonstrated an increased risk of benign paroxysmal positional vertigo as well as its recurrence in individuals with diabetes mellitus [8,22,23]. However, certain investigations have not found statistically significant associations between benign paroxysmal positional vertigo, diabetes, or its recurrence [24,25,26]. The exact mechanism behind the detachment of otoconia—small calcium carbonate crystals in the inner ear responsible for benign paroxysmal positional vertigo—in diabetes remains unclear. In patients with type 1 diabetes mellitus, there was a significantly higher prevalence of deposits in the semicircular canals; this suggests that changes to endolymphatic glucose levels may lead to detachment of otoconia from the saccule and utricle [27]. On the other hand, hypertension may mediate a higher prevalence of benign paroxysmal positional vertigo among patients with type 2 diabetes mellitus by causing vascular damage to the maculae of both utricles and saccules, resulting in dislodgment of otoliths [22]. Nevertheless, our model was adjusted for hypertension, so it remains uncertain what actually causes floating particles within semicircular canals amongst people with type 2 diabetes mellitus. Nonetheless, diabetes mellitus could result in transient ischemia within the inner ear via mechanisms such as microangiopathy or macroangiopathy affecting anterior/posterior vestibular arteries, which supply different parts within the inner ear. Ischemia on maculae located on utricles and saccules could ultimately cause degeneration followed by detachment of otolithic debris, leading to development of benign paroxysmal positional vertigo [28,29]. Further research is needed to better understand underlying mechanisms and pathogenesis associated with benign paroxysmal positional vertigo amongst people living with diabetes mellitus.

### 4.3. Diabetes Mellitus Is Associated with Unilateral Vestibulopathy

Our investigation has uncovered a noteworthy correlation between unilateral vestibulopathy and diabetes mellitus. Multiple other studies have similarly identified diabetes as a significant comorbidity of unilateral vestibulopathy [9,30]. However, one study discovered that the prevalence of diabetes mellitus was no higher in patients with superior unilateral vestibulopathy than in the general population [31]. Unilateral vestibulopathy is believed to result from damage to the vestibular nerve, which may stem from various causes including infection, vascular issues, and immunological factors [32]. In animal models, diabetes mellitus has been demonstrated to induce abnormalities in myelin sheaths within the vestibular nerves. These anomalies include osmiophilic inclusion bodies, disrupted myelin sheath lamellae, lysosomal bodies, periaxonal expansions of Schwann cell cytoplasm [33], and thinner myelin sheaths in horizontal canal nerves [34]. Such changes can arise due to impaired oxygen and nutrient supply to the nerves or breakdown of myelin caused by excessive nonenzymatic glycosylation. Additionally, microangiopathy related to diabetes mellitus leads to an increase in capillary diameters and vascularization of both saccule and utricle [35]. Degenerating type I hair cells scattered throughout the saccular neuroepithelium further suggest reduced oxygen delivery to tissues [36]. Moreover, lower density of type I vestibular hair cells was observed in human temporal bones’ neuroepithelial pathology present within subjects with diabetes’s saccules [37]. Based on these observations, we propose that vascular alterations induced by diabetes mellitus may contribute towards the development of unilateral vestibulopathy. However, it is worth note that diabetes is also a significant vascular risk factor for developing posterior circulation ischemia, which often mimics the presentation of unilateral vestibulopathy [38]. Further research is necessary to fully understand the underlying mechanisms while confirming the role played by vascular changes when diagnosing individuals suffering from diabetic-related unilateral vestibulopathy.

### 4.4. Diabetes Mellitus Is Associated with Peripheral Vestibular End Organ

Our study demonstrated a correlation between diabetes mellitus and peripheral vestibular disorders, with varying findings across the literature. While most studies support the notion that diabetes mellitus can cause vestibular dysfunction or involve the vestibular end organs [7], some studies have specifically shown diabetes mellitus to induce asymptomatic vestibular dysfunction [39,40]. Conversely, other studies have not found any significant association between diabetes mellitus and vestibular dysfunction [7,41]. One proposed mechanism suggests that hypoglycemia triggers the formation of reactive oxygen species, activating the polyol and protein kinase C pathway. This pathway subsequently leads to an overproduction of extracellular matrix, increased lipid droplets, and lysosomes in the connective tissue of the utricle and saccule. Consequently, oxygenation, nutrient supply, as well as waste removal in maculae of both utricle and saccule become impaired, resulting in degeneration of type 1 hair cells [36,37]. Another manifestation of hypoglycemia involves glycosylation of myelin, leading to advanced glycation end products formation, which ultimately causes lysosomal digestion, affecting significant portions of vestibulocochlear nerve. Thinning of myelin sheaths along with reduced axonal fiber diameters further contributes towards longer latency characterized by reduced amplitude on vestibular evoked potentials [3], ultimately resulting in vestibular dysfunction. In order to fully comprehend underlying mechanisms along with clinical implications regarding association between diabetes mellitus and peripheral vestibular disorders, more research is mandatory at this point.

This study exhibits its prowess in multiple domains. Firstly, it enjoys a significant advantage by utilizing an extensive and real-world sample of patients derived from a nationwide population-based dataset. This methodology ensures that the results are representative of the general population and can be applied to a broader context. Furthermore, this study leverages the strengths of the Taiwanese healthcare system, which is not only easily accessible but also affordable for all residents. This comprehensive system with low copayments minimizes bias based on socioeconomic status or residential location, resulting in a more diverse patient sample. Another notable strength lies in its ability to collect data on diabetes and peripheral vestibular diseases from all available sources through NHI claims data that cover healthcare utilization for all Taiwanese residents. This comprehensive and affordable healthcare system ensures that individuals with various medical conditions ranging from minor to severe such as diabetes and vertigo can seek prompt medical attention without being hindered by socioeconomic factors. Consequently, recognition of diabetes mellitus and peripheral vestibular disorders remains unaffected by these factors, leading to more accurate and reliable data. Additionally, leveraging NHI claims data effectively sidesteps potential recall bias often present in self-reported survey data; lastly, this study design implements a case-control approach, with controls selected through propensity score matching, further reinforcing credibility while enabling causal inference between diabetes and vertigo while minimizing selection misclassification bias.

Despite its strengths, it is essential to acknowledge the limitations of this study. A significant constraint lies in the inherent vulnerability of NHI claim data to coding inaccuracies, a common issue with claims data coded by various physicians, such as neurotologists, otolaryngologists, emergency doctors, and primary care doctors, following different examinations. Although the study utilized recorded codes for peripheral vestibular disorders based on widely used medical diagnosis standards (ICD-9-CM and ICD-10-CM codes), these codes may not be as precise as diagnoses determined through standardized clinical examinations by the same specialist team. This lack of standardization introduces the possibility of misclassification bias, such as misdiagnosing central vertigo as peripheral vertigo in certain cases. Furthermore, even experienced doctors may find it challenging to differentiate between some vestibular disorders without further detailed examinations. Since the study relied solely on the coding system and lacked patient follow-up information, additional bias might be present. Nevertheless, the researchers contend that any misclassification bias is likely to be random and improbable to significantly impact the validity of their findings. Another limitation pertains to absent data on confounding variables comprising family history, environmental factors (e.g., stressors, lifestyle choices, or diets), minor head trauma-related genetic factors, race, and laboratory related diabetes mellitus and vertigo information. While this research benefits from being population-based with a large and diverse sample size, these unaccounted variables could potentially influence the relationship between diabetes mellitus and vertigo, thereby restricting generalizability across regions or countries.

## 5. Conclusions

Our study found an association between diabetes mellitus and peripheral vestibular disorders. We have illustrated that patients with diabetes mellitus may experience peripheral vestibular disorders. It is imperative for the physician to recognize the potential presence of an otoneurologic element in these individuals.

## Figures and Tables

**Table 1:** Demographic characteristics and medical co-morbidities of sampled patients classified by peripheral vestibular disorders in Taiwan (*n* = 603,664).

Variable	Patients with Peripheral Vestibular Disorders (*n* = 150,916)		Controls (*n* = 452,748)		*p*-Value
	Total No.	%	Total No.	%	
Age, mean (SD)	56.4 (16.9)		56.4 (16.9)		0.653
Males	58,260	38.6	174,780	38.6	>0.999
Monthly Income **					0.002
<NT$1~15,841	28,289	18.7	84,897	18.8	
NT$15,841~25,000	55,617	36.9	164,693	36.4	
≥NT$25,001	67,008	44.4	203,158	44.8	
Geographic Region ***					<0.001
Northern	73,233	48.5	222,189	49.1	
Central	34,806	23.1	104,449	23.1	
Southern	39,285	26.0	117,354	25.9	
Eastern	3502	2.4	8756	1.9	
Urbanization Level **					0.003
1 (most urbanized)	41,557	27.5	126,541	28.0	
2	43,861	29.1	131,563	29.1	
3	26,590	17.6	79,889	17.6	
4	19,620	13.0	58,066	12.8	
5 (least urbanized)	19,288	12.8	56,689	12.5	
Hypertension	66,717	44.2	200,151	44.2	>0.999
Coronary Heart Disease	29,346	19.5	88,038	19.5	>0.999
Hyperlipidemia	64,140	42.5	192,420	42.5	>0.999

Note: *p*-values were calculated by chi-square tests and *t*-tests; PVDs = peripheral vestibular disorders; ** *p* < 0.01; *** *p* < 0.001.

**Table 2:** Prevalence of diabetes among patients with peripheral vestibular disorders relative to controls.

Variable	Presence of Diabetes *n* (%)
Patients with peripheral vestibular disorders (*n* = 150,916)	31,124 (20.6)
Patients with Ménière’s disease	4396 (22.5)
Patients with benign paroxysmal positional vertigo	4294 (20.7)
Patients with unilateral vestibulopathy	2637 (20.6)
Patients with other peripheral vestibular disorders	19,797 (20.2)
Controls (*n* = 452,748)	68,032 (15.1)

**Table 3:** Crude and covariate-adjusted odds ratios for diabetes among patients with peripheral vestibular disorders (PVDs) and controls.

Variable	Presence of Diabetes	
	OR (95% CI)	Adjusted OR ^a^ (95% CI)
Patients with peripheral vestibular disorders	1.463 * (1.441~1.485)	1.597 * (1.570~1.623)
Patients with Ménière’s disease	1.443 * (1.387~1.502)	1.566 * (1.498~1.638)
Patients with benign paroxysmal positional vertigo	1.522 * (1.462~1.585)	1.677 * (1.603~1.755)
Patients with unilateral vestibulopathy	1.466 * (1.393~1.543)	1.592 * (1.504~1.685)
Patients with other peripheral vestibular disorders	1.454 * (1.428~1.482)	1.588 * (1.555~1.621)
Controls	1.000	1.000

Notes: CI = confidence interval; OR = odds ratio; ^a^ Adjusted for age, monthly income, geographic location, urbanization level, hyperlipidemia, coronary heart disease, and hypertension. * *p* < 0.001.

## Data Availability

The LHID2010 can be obtained by interested researchers through a formal application process addressed to the HWDC, Department of Statistics, Ministry of Health and Welfare, Taiwan https://dep.mohw.gov.tw/DOS/lp-2506-113.html (accessed on 21 December 2023).
